# Benchmarking dataset for leak detection and localization in water distribution systems

**DOI:** 10.1016/j.dib.2023.109148

**Published:** 2023-04-14

**Authors:** Mohsen Aghashahi, Lina Sela, M. Katherine Banks

**Affiliations:** a Texas A&M Institute of Data Science, Texas A&M University, 155 Ireland Street, College Station, TX 77843, USA; b Civil, Architectural and Environmental Engineering, The University of Texas at Austin, 301 E. Dean Keeton St., Austin, TX 78712, USA; c Texas A&M University, 400 Bizzell St., College Station, TX 77843, USA

**Keywords:** Leak, Anomaly detection, Water networks, Sensors, Supervised and unsupervised classification

## Abstract

This paper presents a dataset with two hundred and eighty sensory measurements for leak detection and localization in water distribution systems. The data were generated via a laboratory-scale water distribution system that included (1) three types of sensors: accelerometer, hydrophone, and dynamic pressure sensor; (2) four leak types: orifice leak, longitudinal and circumferential cracks, gasket leak, and no-leak condition; (3) two network topologies: looped and branched; and (4) six background conditions with different noise and demand variations. Each measurement was 30 s long, and the measurement frequencies were 51.2 kHz for the accelerometer and dynamic pressure sensors, and 8 kHz for the hydrophone. This is the first publicly available dataset for advancing leak detection and localization research, model validation, and generating new data for faulty sensor detection in water distribution systems.

**Specifications Table**

**Table:** 

Subject	Water Science and Technology
Specific subject area	Leak simulations in experimental testbed water networks
Type of data	Tabular Sound
How the data were acquired	Signals were acquired by: (1) Sensors: two accelerometers (PCB 333B50), two hydrophones (Aquarian H2c), and two dynamic pressure sensors (PCB 102B16); (2) Data acquisition instruments (DAQs): NI-9234 for accelerometer and dynamic pressure data and ZOOM UAC-2 audio converter for hydrophone data; (3) Software: LabVIEW NXG 5.1 for accelerometer and dynamic pressure sensor data, and Audacity 3.0.5 for hydrophone data.
Data format	Raw
Description of data collection	The dataset was generated through controlled leak experiments in a laboratory-scale (lab) water distribution testbed with 152.4 mm diameter PVC pipes and 47 m total pipe length. The following factors were changed in the experiments: network topology (looped and branched), leak type (orifice, longitudinal, circumferential, and gasket) and no-leak condition, background flow (0, 0.18, 0.47 L/s, and transient with an abrupt flow change from 0.47 to 0 L/s), background noise (traffic and tool noise), and using three different types of sensors (accelerometer, hydrophone, dynamic pressure). Overall, two hundred and eighty signals were recorded, where the length of each signal was 30 s. Signals were saved on a local computer via low-noise cables and the abovementioned DAQs and software.
Data source location	Institution: Texas A&M University City/State: College Station, Texas Country: United States Latitude and longitude for collected samples/data: 30°36′37.33″ N −96°20′38.60″ W
Data accessibility	Repository name: Mendeley Data Data identification number: DOI: 10.17632/tbrnp6vrnj.1 Direct URL to data: https://data.mendeley.com/datasets/tbrnp6vrnj/1*.*[1]

## Value of the Data

• This is the first fully labeled and publicly available dataset for leak detection and localization in water distribution systems (WDSs). The data generated by controlled experiments enable the research community to evaluate how hydraulic and physical factors affect leak footprints based on sensory measurements. In addition, the data can be used for: (1) developing and validating algorithms for leak detection and localization, (2) evaluating the sensitivity and feasibility of the three different types of sensors for leak detection, and (3) identifying significant features and covariates for machine learning algorithms for leak detection.
• The data can be utilized by (1) hydroinformatics researchers for developing and testing machine learning algorithms for leak detection and localization, (2) applied machine learning researchers that need a benchmark dataset for model validation, and (3) cybersecurity professionals researching anomaly detection in sensory data.
• The data can be further modified by, for example, adding noise or missing values and be used to develop anomaly detection algorithms with data from noisy or faulty sensors. Moreover, the data can be augmented by resampling and utilized as on-the-fly data points for validating online learning algorithms. Testbed specifications can also be used to develop new testbeds for leak simulation and design new experiments to accommodate different conditions.

## 1 Objective

Leaks waste millions of liters of treated water in water distribution systems around the globe. There is extensive ongoing research for leak detection using various sensors, e.g., flow, pressure, hydrophone, and accelerometers in lab testbeds. We identified several gaps in the previous literature: (1) most lab testbeds have several limitations, such as small pipe diameters, single pipelines, lack of complicated network topology, not accounting for background noise and flow conditions, and simulating only orifice leak type; and (2) none of the studies have made the recorded data available to researchers, thus limiting further algorithmic development and testing. To address these gaps, we designed and built a comprehensive lab testbed addressing the abovementioned limitations and recorded data from multiple leak experiments measured by different sensors. This paper aims to provide the data to the research community to promote research usability and reproducibility.

## 2 Data Description

The data is arranged in three folders: Accelerometer, Hydrophone, and Dynamic Pressure Sensor, which include the recorded signals corresponding to each sensor and each leak experiment. Each sensor folder includes data measured at WDSs with two topologies, i.e., branched and looped, where four leak types (circumferential crack, longitudinal crack, orifice leak, and gasket leak) and no-leak were simulated. The Hydrophone folder also contains the Background Noise folder, which includes two files: "Background Noise_H1″ and "Background Noise_H2". These files are the measurements of one generated background noise recorded by hydrophones H1 and H2 at different locations. The "Background Noise_H1″ and "Background Noise_H2″ signals only include a saw and traffic sounds recorded simultaneously, while there were no pump, flow, and leak sounds in the measurements. Measurement units for accelerometer, dynamic pressure and hydrophone data are m/s2, Pascal (Pa), and Volt (V), respectively. The unit of the hydrophone data can be converted to decibels (dB) via *S* = 20 log10|*X*|, where S is sound intensity (dB), and *X* is the hydrophone measurements (V).

**Fig. 1:**
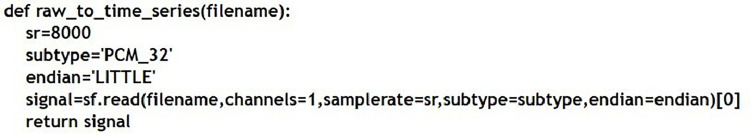
Code to read raw hydrophone data in Python.

**Fig. 2:**
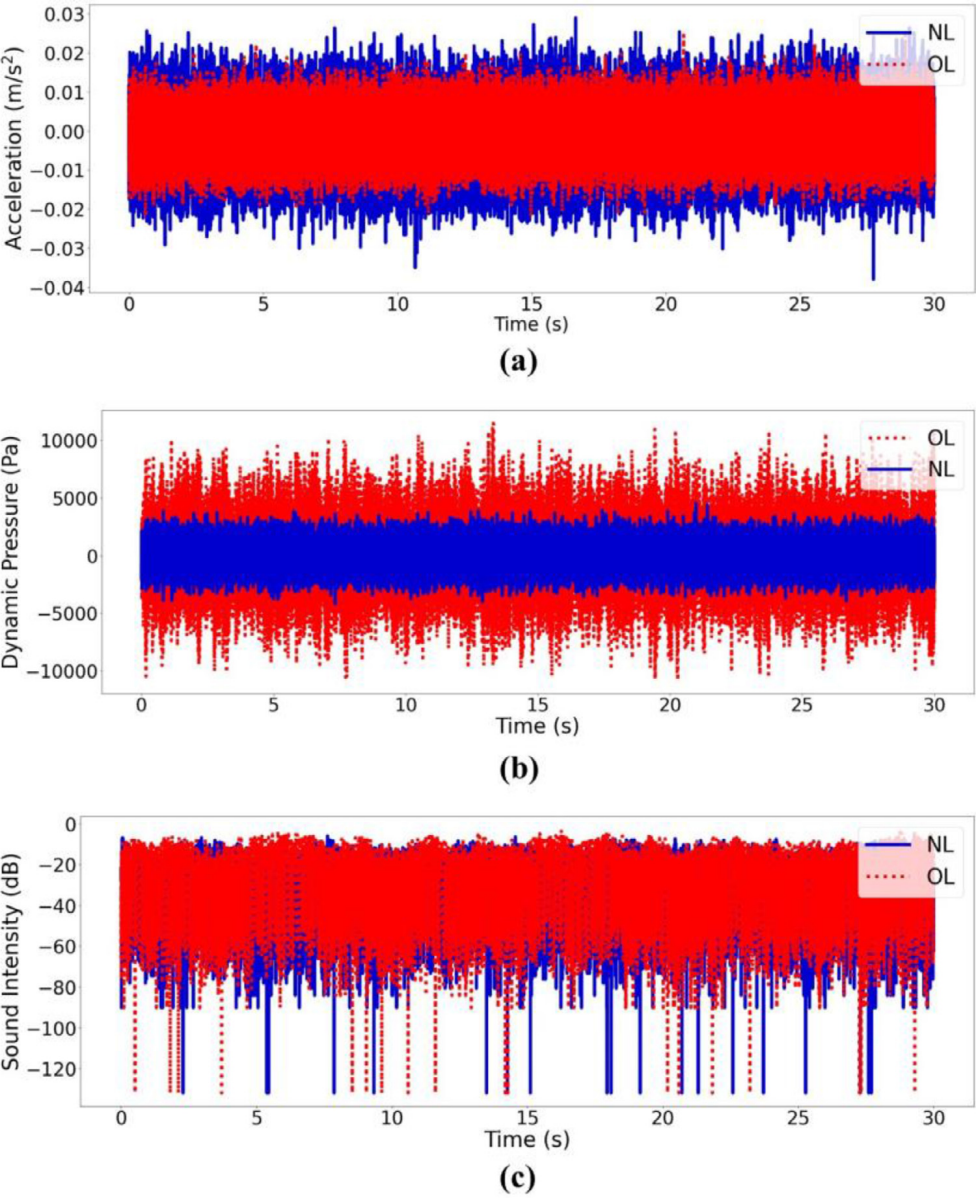
Example of data recorded by the sensors with 0.18 L/s demand, NL (in solid line), and OL (in dotted line) measured by (a) accelerometer A1, (b) dynamic pressure sensor P1, and (c) hydrophone H1 (NL: no-leak; OL: orifice leak).

Accelerometer and dynamic pressure sensor data are recorded in ’.csv’ format and the files are labeled as *“T_L_F_S#”*, where *T* represents network topology, *L* leak type, *F* background flow condition, *S* sensor type, and *#* sensor number. Table 1 lists all the acronyms in the names of the data files. For example, *"BR_CC_0.18 LPS_A1″*is a signal recorded at the branched network with a circumferential crack, 0.18 liters per second (LPS) demand, and measured by accelerometer number 1. Hydrophone data files in the Branched and Looped folders are named as *“T_L_F_B_ S#”* where *T, L, F, S* and *#* represent the same parameters as those of the accelerometer and dynamic pressure data, and *B* indicates background noise conditions. For instance, *“LO_OL_ND_N_H2”* is a signal recorded at the looped network with an orifice leak, no demand (0 L/s), the background noise present, and measured by hydrophone number 2. Acoustic data in the Hydrophone folder are in ‘.raw’ format that needs to be converted to time series data before analysis. Fig. 1 shows a snippet of a Python code that reads a raw data file and returns a time series. As an example, Fig. 2(a)–(c) display accelerometer *A1*, dynamic pressure sensor *P1*, and hydrophone *H1* signals measured at the looped network with 0.18 L/s demand, orifice leak and no-leak conditions, and background noise present.

**Table 1:** Summary of different conditions and their acronyms.

Category	Term	Definition
Network topology	BR	Branched network
	LO	Looped network

Leak type	CC	Circumferential crack
	LC	Longitudinal crack
	GL	Gasket leak
	OL	Orifice leak
	NL	No-leak

Sensor type	A	Accelerometer
	H	Hydrophone
	P	Pressure sensor

Background noise	N	With background noise
	NN	Without background noise

Background flow condition	ND	No demand (0 L/s)
	0.18 LPS	0.18 L/s demand
	0.47 LPS	0.47 L/s demand
	T	Transient - demand abruptly changed from 0.47 L/s to 0 L/s at approximately second 20 after the start of the leak experiment.

## 3 Experimental Design, Materials and Methods

In this section, we describe the main stages of our testbed: Phase 1 – includes the experimental setup, components, and layout; Phase 2 – describes leak characteristics, shapes, and magnitudes; Phase 3 – provides sensor specifications; Phase 4 – describes sensor specifications and locations; Phase 5 – explains the data acquisition procedure; and Phase 6 – summarizes the various leak experiments that were conducted.

### 3.1 Phase 1: Experimental Setup

**Fig. 3:**
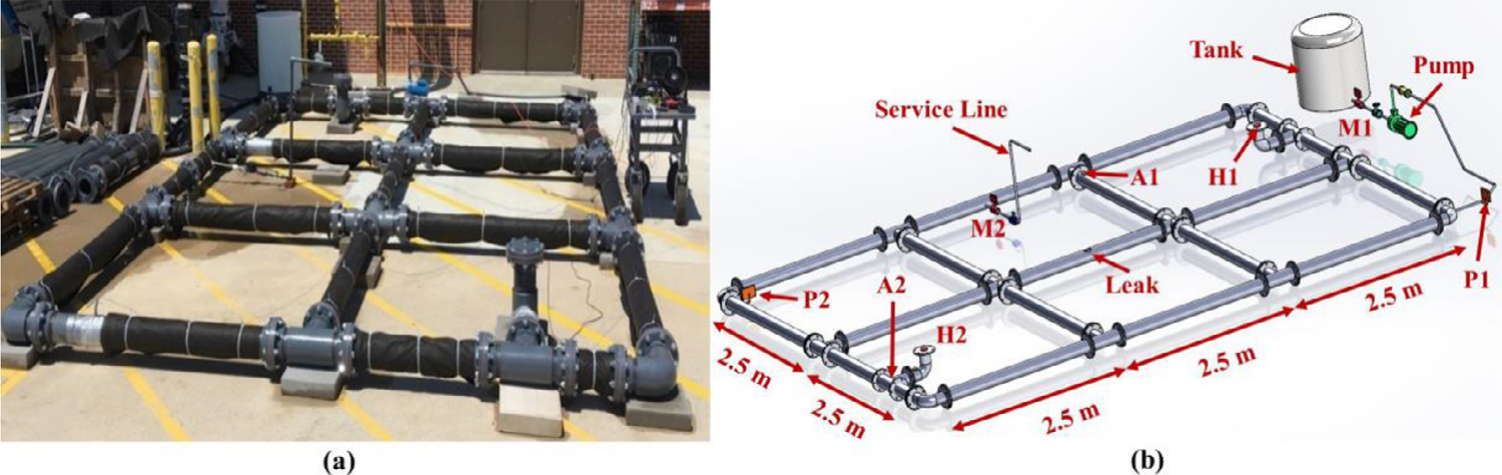
(a) A picture and (b) a schematic of the looped network (A: accelerometer; H: hydrophone; P: dynamic pressure sensor; M: meter).

**Fig. 4:**
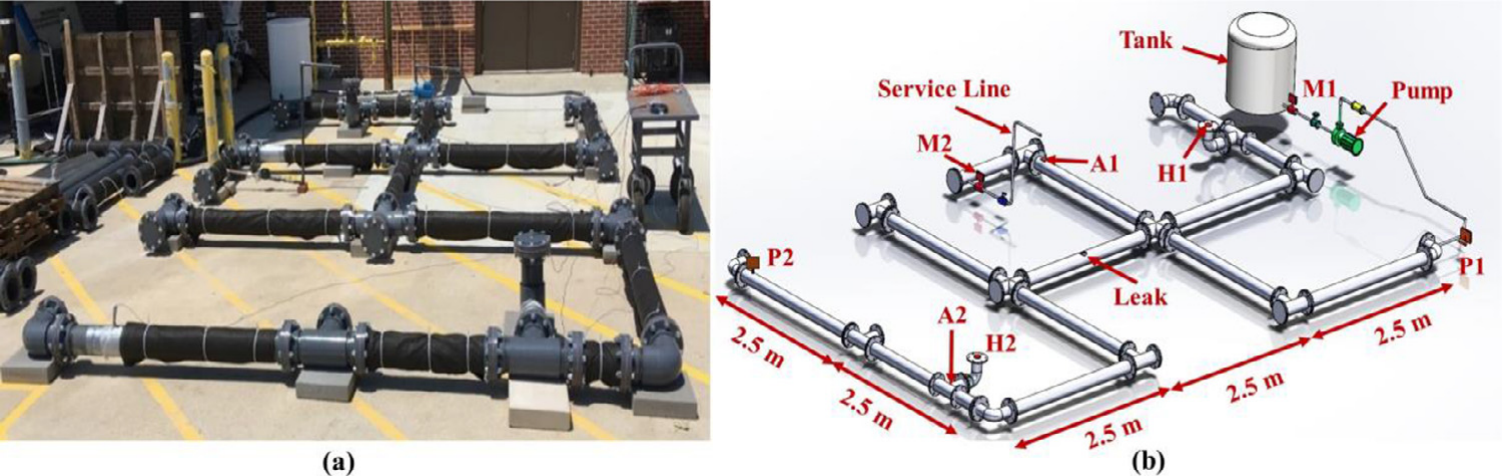
(a) A picture and (b) a schematic of the branched network (A: accelerometer; H: hydrophone; P: dynamic pressure sensor; M: meter).

***Network Layout.*** The testbed was 7.5 m long, and 5 m wide, with 47 m total pipe length, and included a water supply line and a distribution section. The supply line provided pressurized water to the distribution section. The distribution section comprised: (1) seventeen 152.4 mm schedule 80 PVC pipes reconfigured in looped and branched topologies, (2) two prototype hydrants, each 355.6 mm in height built from 152.4 mm diameter PVC pipe, (3) one service line with the height of 1092.2 mm to simulate demand. Pipes were connected to fittings via flanges, enabling us to reconfigure the layout of the network into looped and branched topologies. Fig. 3, Fig. 4 show the testbed with the looped and branched layouts, and locations of the hydrants, sensors, and service line.

**Fig. 5:**
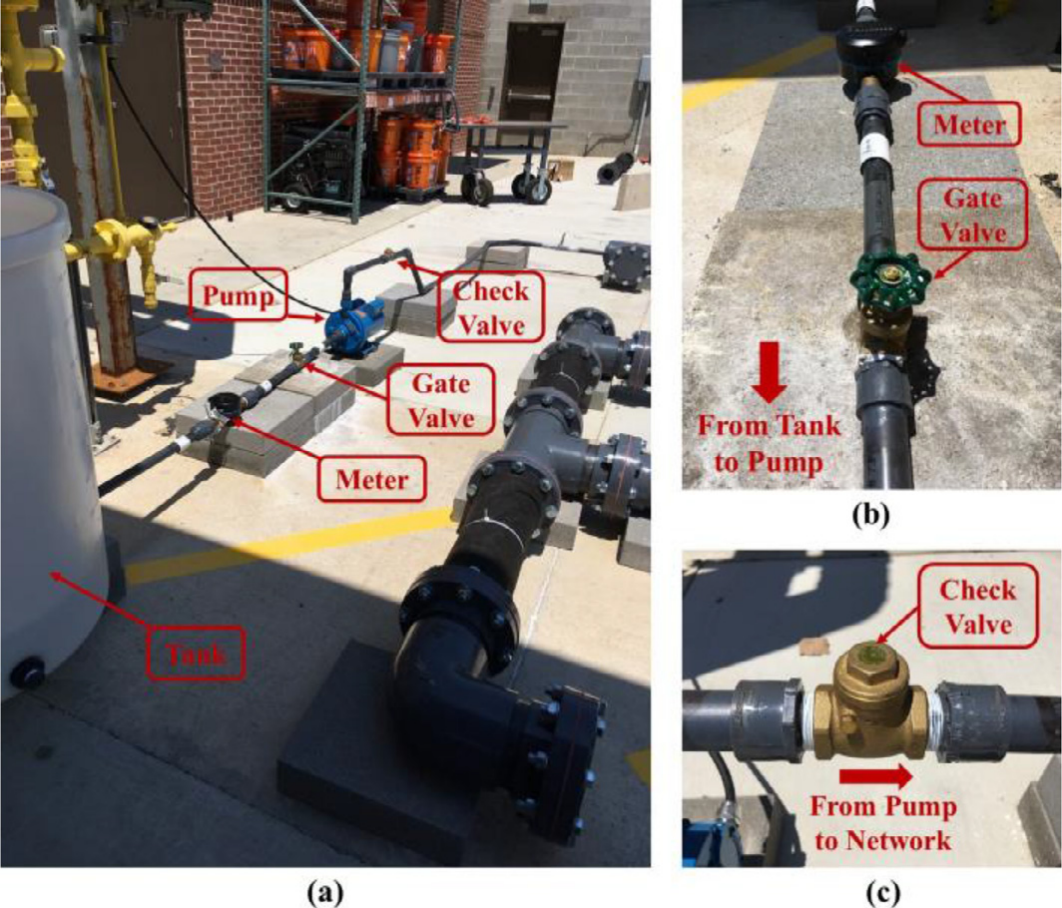
(a) An overview of the water supply line, (b) meter and gate valve, and (c) check valve.

***Water Supply Line.*** The 25.4 mm diameter supply line included a storage tank, a flow meter, a gate valve, a pump, and a check valve. Fig. 5 shows the supply line and its components. Three reducers were used to connect the 25.4 mm supply line to a 152.4 mm pipe of the distribution section. We used the fewest fittings possible and employed 45-degrees elbows to decrease minor head losses. The storage tank was a plastic open-top cylinder with a height of 920 mm and a diameter of 800 mm, filled with a water hose. For measuring the total input water to the distribution section, we used a 25.4 mm Neptune MACH 10 ultrasonic meter with a 0.0038 L resolution. The Matco brass gate valve in Fig. 5 was used to adjust the water flow into the distribution section and control the input water volume. A fixed-speed centrifugal pump, Goulds 1MC1G1A0, with a maximum of 43 m cut-off head and a maximum of 15 m^3^/s flow rate, was used to supply water from the tank to the distribution section. Finally, a Matco brass check valve was installed to prevent backflow from the distribution section to the pump.

***Support Blocks.*** Due to the water pressure in the pipes and resulting momentum at the junctions, we put 22.68 kg concrete blocks under each fitting to prevent the fittings from moving, as shown in Fig. 3(a) and Fig. 4(a). With all fittings constrained, we could stabilize the entire testbed.

***Backfill Medium.*** To simulate the damping effects of backfill materials on leak frequency and attenuation rates, all pipes were covered with two layers, 50 mm thick, of Mutual NW100 non-woven geotextile fabric. This type of fabric provides a good representation of unfluidized media surrounding pipes in lab testbeds [2].

**Fig. 6:**
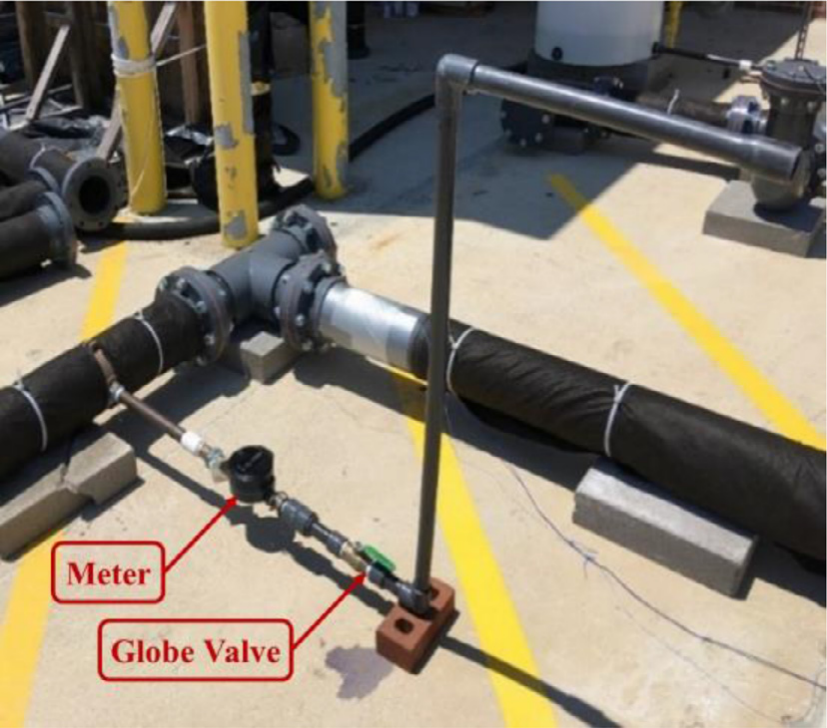
Service line with meter and globe valve.

***Background Flow Conditions.*** A service line with a 25.4 mm diameter pipe was connected to the distribution section with a saddle clamp to simulate the background flow conditions. Background flow conditions were simulated assuming that the distribution section supplied water demand for approximately 100 people, which resulted in an approximate 0.44 L/s total water demand [3]. Then, to account for daily variability in demand, we used 0.41 and 1.06 multipliers representing demands at 1:00 am and 5:00 am, respectively, resulting in 0.18 L/s and 0.47 L/s water demands. The reason for choosing 1:00 am and 5:00 am multipliers is the relatively low water consumption and low background noise at these hours [4]. We also simulated a no-demand condition, i.e., 0 L/s, to evaluate the effects of background flow on leak signals. In the no-demand condition, there was no outflow from the service line, and only leak and no-leak conditions determined if there was water flow in the pipes. In the leak condition, water exiting from the location of the leak was the only outflow from the testbed. In the no-leak condition, water was standing in pipes, and there was no outflow from the testbed. In addition, we simulated transient conditions, in which the flow rate was abruptly changed from 0.47 L/s to 0 L/s by rapidly shutting off the globe valve on the service line approximately second 20 after the start of the leak experiment. Fig. 6 shows the service line, which includes a Neptune MACH 10 ultrasonic meter to measure the demand flows, and a globe valve to adjust the outflow of the service line.

***Background Noise.*** Background noise was generated by simultaneously playing a traffic sound using a speaker located at the center of the testbed and using an electric saw that was moved around the testbed to different locations while measurements were taken. The background noise was generated based on device availability and similar to real conditions, in which sounds of traffic and rotating machinery may mask leak signals and add noise to the acoustic data [5,6]. Since the location of the saw was changed randomly, the background noise is expected to be dissimilar not only at different points of the testbed but also throughout the duration of the measurements.

### 3.2 Phase 2: Leak Characteristics

***Leak Type.****We conducted* experiments with four types of leaks: orifice leak (OL), longitudinal crack (LC), circumferential crack (CC), and gasket leak (GL). These leaks were induced in the middle pipe of the testbed, as shown in Fig. 3(b) and Fig. 4(b). We induced the orifice and cracks by drilling and milling the middle pipe wall, respectively. The gasket leak was induced by loosening a flange's bolts located in the middle of the leaking pipe. It is worth noting that the leaks were induced in four different pipes, and experiments were conducted one at a time for each type. Also, for generating data with no leak, we conducted experiments with a leak-free pipe that simulated no-leak (NL) conditions.

**Fig. 7:**
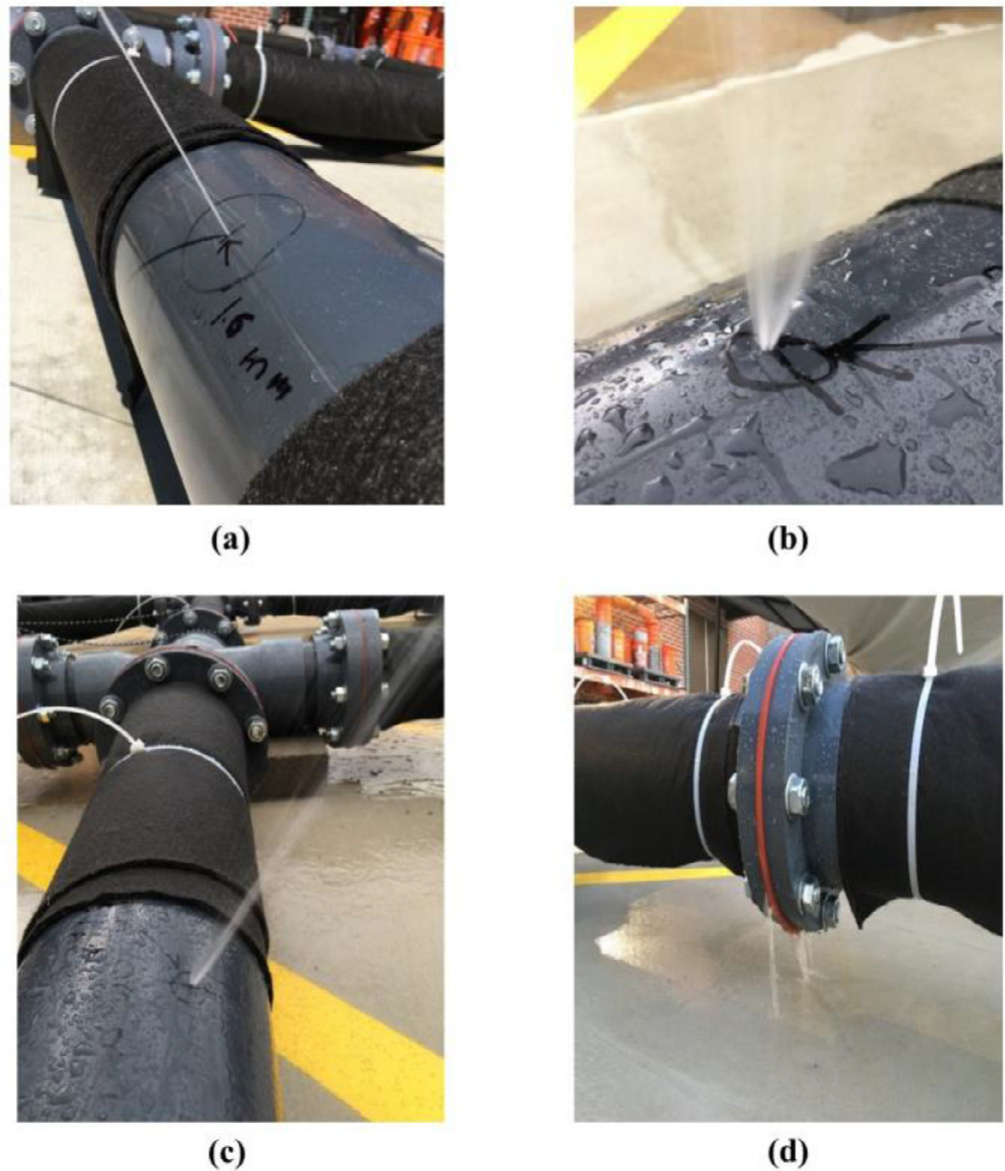
Water jets from (a) orifice leak (OL); (b) longitudinal leak (LC); (c) circumferential leak (CC); and (d) outflow from gasket leak (GL).

Fig. 7 shows the OL, LC, CC, and GL and their water jets and outflow during leak experiments.

**Fig. 8:**
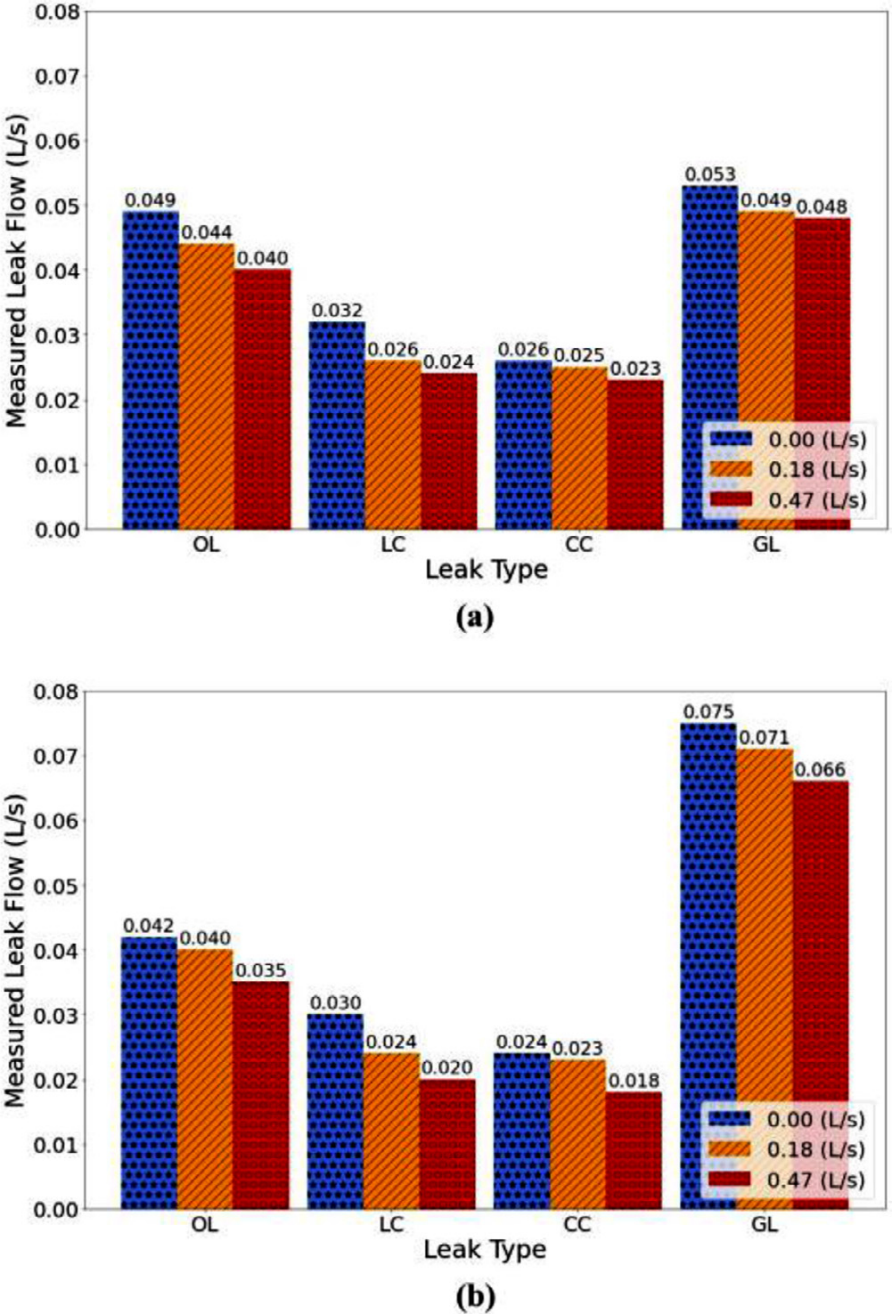
Measured leak flows with different leak types and demands: (a) looped and (b) branched network.

**Leak Size and Flow.** In this work, we aimed to induce leaks with flows less than 30% of the total input water to the testbed. Based on our available equipment, we created a 2 mm2 leak area and used milling to create longitudinal and circumferential cracks with the dimensions of 2 mm × 1 mm and drilling to induce an orifice with a diameter of about 1.6 mm. Our initial calculations showed that 2 mm^2^ could generate leak flows smaller than 30% of the total input water to the testbed. The actual leak flow rate generated in each experiment was computed by calculating the difference between the measured flow entering the system through the supply line and the measured flow leaving the system through the service line. Fig. 3 and Fig. 4 show the locations of the flow meters M1 and M2 located on the supply and service lines, respectively. For each experiment with a different network topology and a different leak type, we simulated different flow conditions by manually adjusting the globe valve located on the service line to reach the desired flow rate conditions. As described earlier, three flow conditions were tested, 0, 0.18, and 0.47 L/s flow through the service line. We measured leak flows to evaluate how demand flows affected leak flows. In addition, we compared outflow shapes and flow rates for different leak types. Table 2 lists the simulated demand, the flow rates measured by meters M1 and M2, the leak flow rates, and the percentage of water loss in the system through leaks (%), calculated as the ratio between the leak flow and the total flow entering the system, for the looped and branched network topology, and each leak type. We observe that all leaks generated in these experiments were below 30% of input flow, satisfying our leak flow rate objective. Fig. 8(a) and Fig. 8(b) show the measured leak flows in the looped and branched networks, respectively, where numbers represent the measured leak flows rates.

### 3.3 Phase 3: Sensor Characteristics

We deployed two hydrophones, two accelerometers, and two dynamic pressure sensors. Accelerometers measured pipes’ vibration, and hydrophones and dynamic pressure sensors measured sounds and pressure variations inside the pipes.

Table 3 includes the characteristics of the sensors. Sensors were selected so that (1) accelerometers could measure limited vibrations generated by small leaks, (2) hydrophones could capture a spectrum of leak frequencies with enough sensitivity, and (3) dynamic pressure sensors could detect small leaks with subtle pressure changes. The hydrophones had low self-noise and incorporated a matched sensor and Field Effect Transistor Circuit Design (FET) buffer amplifier assembly that produced an output electrically equivalent to electret-condenser microphones powerful enough to amplify small leak sounds. The accelerometers had ceramic sensing elements, a resonant frequency ≥20 kHz, a frequency range of 0.5 to 3000 Hz, and a temperature range of −18 to +66 °C. The pressure sensors had quartz sensing elements, a measurement resolution of 0.007 kPa, a resonant frequency ≥500 kHz, and a temperature range of −73 to +135 °C. These specifications of the sensors made them suitable to capture leak signals with high resolution and low noise.

### 3.4 Phase 4: Sensor Locations

**Fig. 9:**
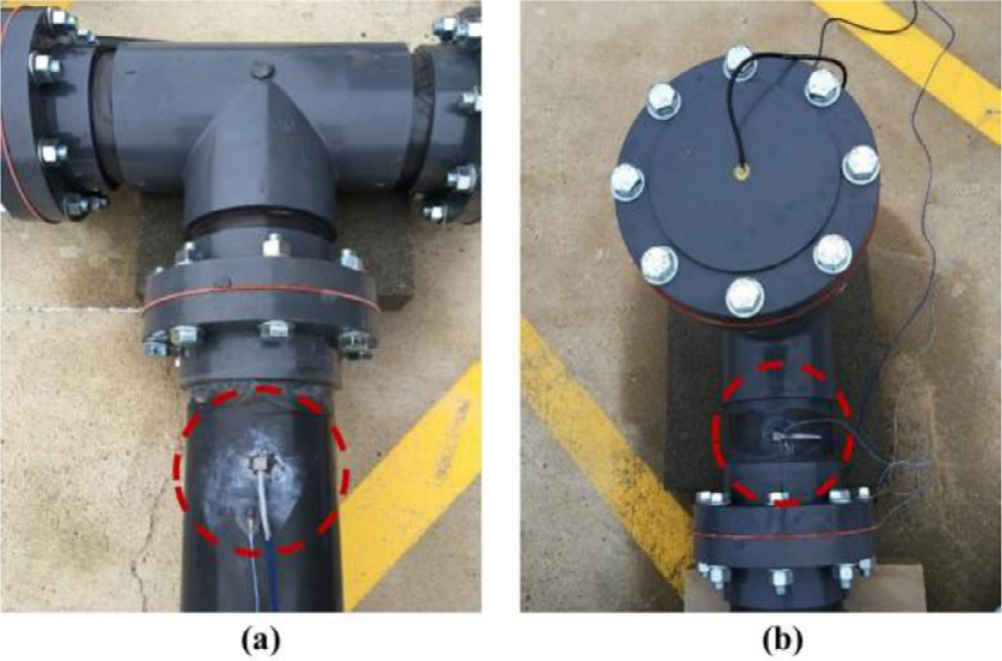
Accelerometers: (a) A1 located on a leg of a tee connection and (b) A2 located on a leg of the tee connection at the hydrant with hydrophone H2. The dashed circles pinpoint the locations of the accelerometers.

***Accelerometer Locations.*** Accelerometers require direct contact with the pipe and, hence, are typically installed at valve access points [7,8]. According to valve design guidelines, valves should be located at (1) two branches of a tee connection, and (2) at the end of a service line [3]. We installed accelerometers A1 and A2 on the branches of two different tee connections (see Fig. 3(b) and Fig. 4(b)). Fig. 9(a) and Fig. 9(b) show pictures of A1 and A2 locations, where the dashed circles pinpoint the locations of the accelerometers.

**Table 2:** Measured leak flows with different network topology, leak types, and demands.

Topology	Leak Type	Design Demand (L/s)	Flow M1 (L/s)	Flow M2 (L/s)	Measured Leak Flow (L/s)	Leak Rate (%)
Looped	OL	0.00	0.049	0.000	0.049	–
		0.18	0.237	0.193	0.044	19
		0.47	0.512	0.472	0.040	8
	LC	0.00	0.032	0.000	0.032	–
		0.18	0.222	0.196	0.026	12
		0.47	0.497	0.473	0.024	5
	CC	0.00	0.026	0.000	0.026	–
		0.18	0.214	0.189	0.025	12
		0.47	0.498	0.475	0.023	5
	GL	0.00	0.053	0.000	0.053	–
		0.18	0.240	0.191	0.049	20
		0.47	0.524	0.476	0.048	9

Branched	OL	0.00	0.042	0.000	0.042	–
		0.18	0.239	0.199	0.040	17
		0.47	0.510	0.475	0.035	7
	LC	0.00	0.030	0.000	0.030	–
		0.18	0.222	0.198	0.024	11
		0.47	0.495	0.475	0.020	4
	CC	0.00	0.024	0.000	0.024	–
		0.18	0.216	0.193	0.023	11
		0.47	0.494	0.476	0.018	4
	GL	0.00	0.075	0.000	0.075	–
		0.18	0.265	0.194	0.071	27
		0.47	0.541	0.475	0.066	12

**Table 3:** Characteristics of the sensors deployed in the testbed.

Sensor	Brand	Model	Sensitivity	Measurement Range
Hydrophone	Aquarian	H2c	−180 dB (reference: 1 V/*μ*Pa)	1–100 kHz
Accelerometer	PCB	333B50	102 mV/(m/s²)	±49 m/s² pk
Dynamic pressure	PCB	102B16	7.25 *μ*V/Pa	1–689 kPa

**Fig. 10:**
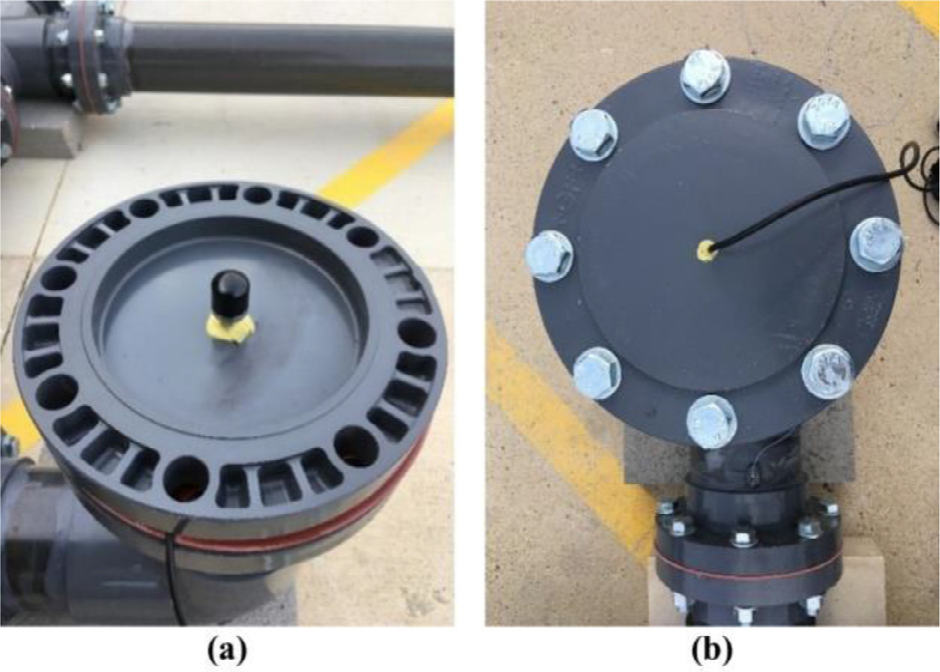
Hydrophones: (a) inside view of the blind flange and (b) top view of the blind flange in the prototype hydrant.

***Hydrophone Locations.*** Hydrophones require direct contact with water, and hence, are often mounted at the top or the bottom of fire hydrants [9,10]. To simulate a hydrant in our testbed, we used an erected 152.4 mm diameter pipe, with a height of 355.6 mm, whose top was closed by a blind flange, and the bottom was connected to the distribution section by an elbow. Then, a hole at the center of the blind flange was drilled and tap threaded, and the hydrophone was screwed into the hole in the flange. Fig. 10(a) shows an inside view of the blind flange with the Aquarian H2c hydrophone, and Fig. 10(b) shows the top view of the setup with the hydrophone in the blind flange. Based on design guidelines, fire hydrants should be located close to intersections or at specific intervals in residential districts [3]. Therefore, we installed one hydrant in the middle of a pipe to mimic a hydrant location in a residential area, and the second hydrant closer to an intersection. The two hydrophones, H1 and H2, were installed at the two hydrants as far as possible from each other and symmetrical to the leak location (see Fig. 3 and Fig. 4).

**Fig. 11:**
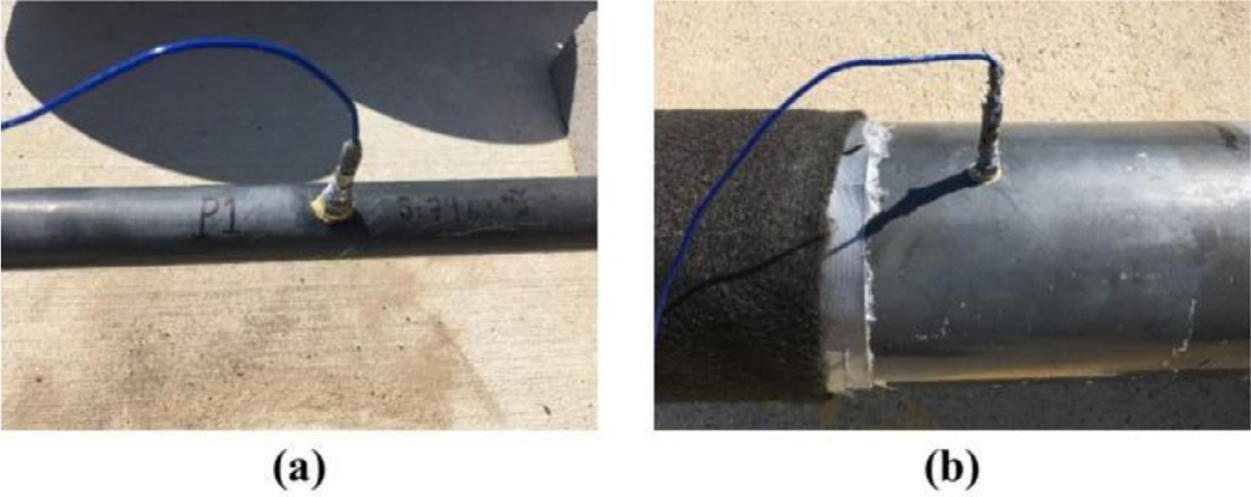
Dynamic pressure sensors: (a) P1 mounted on the supply line and (b) P2 installed in the distribution section.

***Dynamic Pressure Sensor Locations.*** We used two dynamic pressure sensors in the testbed, P1 and P2. P1 was mounted at the end of the supply line (see P1 in Fig. 3(b) and Fig. 4(b)) and measured the dynamic pressure of water before water enters the distribution section. P2 was installed at the farthest corner from the entry point, see P2 in Fig. 3(b) and Fig. 4(b)), and measured dynamic pressure downstream of the leaks. Locations of P1 and P2 enabled us to capture the effects of leaks and network junctions in dynamic pressure measurements. Fig. 11(a) and (b) show P1 and P2 mounted on the supply line and the distribution section, respectively.

### 3.5 Phase 5: Data Acquisition System

Two NI-9234 modules, each with 51.2 kS/s/ch sampling rate and low-noise coaxial cables, were used to acquire the measurements of the dynamic pressure sensors and accelerometers, and LabVIEW NXG 5.1 was used to record the measurements. To acquire hydrophone measurements, the hydrophones were connected to the ZOOM UAC-2 audio converter via low-noise cables. The ZOOM UAC-2 sampled the hydrophone signals via two 24-bit/192 kHz high-resolution input channels. The converter digitized signals and transmitted them to a computer with Audacity 2.3.3 software that recorded the signals in ’.raw’ format at a rate of 8000 Hz and a signed 32-bit pulse-code modulation. All sensory data were recorded for 30 s at rates that follow the Nyquist sampling theorem, where the signal sampling rate must be greater than twice the expected maximum frequency of a signal [7]. Since the expected frequencies of water leak signals are smaller than 1000 Hz [5,11], the sensors and data acquisition system were selected and set with sampling frequencies greater than 2000 Hz.

### 3.6 Phase 6: Experiments

**Fig. 12:**
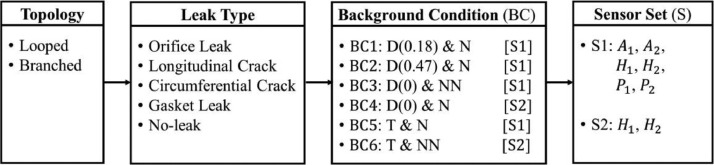
Summary of the simulated scenarios with variations in network topology, leak type, background conditions (i.e., demand and background noise), and sensor type and location (D(x): demand flow where *x* = 0, 0.18, and 0.47 L/s; N: with background noise; NN: without background noise; T: with transient; S: sensor sets).

Having set up the testbed and sensing and data acquisition systems, we performed two hundred and eighty different experiments to test system response to changes in network topology, leak type, background conditions, and combinations of sensors. Fig. 12 shows the different simulated scenarios and Table 1 summarizes all the categories and their acronyms. For each network topology (looped and branched), four leak types (orifice leak, longitudinal crack, circumferential crack, gasket leak) and no-leak scenarios were simulated. For each leak type, different background conditions (BC), i.e., combination of demand and background noise conditions, were generated, resulting in overall six variations: (BC1) 0.18 L/s demand and background noise, (BC2) 0.47 L/s demand and background noise, (BC3) no demand and no background noise, (BC4) no demand and with background noise, (BC5) transient and background noise, and (BC6) transient and no background noise. Demand and no demand conditions specified if the service line had an outflow, and with and without background noise determined whether the background noise, i.e., traffic and saw sounds generated and measured simultaneously, was present. Two different sensor sets were used: (S1) included two of each type of sensor, including hydrophones, dynamic pressure sensors, and accelerometers, and (S2) included two hydrophones. These tests resulted in one hundred and forty recorded signals for each network topology and two hundred and eighty signals in total.

## Ethics Statements

The authors declare that this submission follows the ethical requirements for publication in Data in Brief.

## CRediT authorship contribution statement

**Mohsen Aghashahi:** Conceptualization, Methodology, Writing – original draft, Visualization. **Lina Sela:** Conceptualization, Methodology, Writing – review & editing. **M. Katherine Banks:** Supervision, Funding acquisition.

## Declaration of Competing Interest

The authors declare that they have no known competing financial interests or personal relationships that could have appeared to influence the work reported in this paper.

## Data Availability

Dataset of Leak Simulations in Experimental Testbed Water Distribution System (Original data) (Mendely).
